# A Modified Theoretical Model of Intrinsic Hardness of Crystalline Solids

**DOI:** 10.1038/srep33085

**Published:** 2016-09-08

**Authors:** Fu-Zhi Dai, Yanchun Zhou

**Affiliations:** 1 Science and Technology of Advanced Functional Composite Laboratory, Aerospace Research Institute of Materials & Processing Technology, Beijing 100076, China

## Abstract

Super-hard materials have been extensively investigated due to their practical importance in numerous industrial applications. To stimulate the design and exploration of new super-hard materials, microscopic models that elucidate the fundamental factors controlling hardness are desirable. The present work modified the theoretical model of intrinsic hardness proposed by Gao. In the modification, we emphasize the critical role of appropriately decomposing a crystal to pseudo-binary crystals, which should be carried out based on the valence electron population of each bond. After modification, the model becomes self-consistent and predicts well the hardness values of many crystals, including crystals composed of complex chemical bonds. The modified model provides fundamental insights into the nature of hardness, which can facilitate the quest for intrinsic super-hard materials.

Super-hard materials (H_v_ > 40 GPa) have attracted great attentions because of their practical importance with numerous industrial applications, e.g. cutting and polishing tools, wear resistant coatings, and abrasives^1^,^2^. Over the past several decades, great efforts have been devoted to explore new super-hard materials^1^,^2^,^3^,^4^.

Historically, searching for super-hard materials was guided under correlations between hardness and macroscopic properties, such as bulk modulus *B*^5^ or shear modulus *G*^6^. However, these empirical correlations are physically questionable, which usually result in misleading. Even though Chen *et al.*^7^ elegantly modeled the hardness of a material by introducing the Pugh’s ratio^8^, *k* = *G*/*B*, which characterizes the brittleness/ductility of the material, these macroscopic concepts cannot provide insight into the physical origin of hardness. As a consequence, estimating the hardness directly with microscopic parameters may reveal the fundamental factors that control hardness, which is essential for the design and exploration of new super-hard materials.

Up to now, three categories of microscopic models for hardness evaluation have been proposed^3^,^4^,^9^,^10^,^11^,^12^. All of the microscopic models share the same assumption that the hardness equals to the sum of resistance of each bond per unit area to the indenter^9^ with the resistance estimated under different hypotheses^3^,^4^. The microscopic model proposed by Gao *at al.*^9^ is probably the most popular one with the resistance assumed proportional to the homo-polar energy gap. Later, Gao^10^ suggested that the strength of a bond can be more accurately characterized by using average overlap populations per unit volume of the bond, where the overlap populations is evaluated from first-principles calculations. With the advancement on computational technology, the newly developed formula by Gao^10^ is getting more and more attractive. However, recently, we found that the hardness values of materials with complex bond types estimated from the formula exhibit dramatically discrepancies with experimental measurements. In the present work, we will reexamine the formula in detail and modify it to capture as many materials as possible.

## Theoretical Model and Modifications

In microscopic models, it is usually assumed that any complex crystal can be decomposed into a set of pseudo-binary crystals (chemical bonds), and properties of the crystal can be derived from readily accessible parameters associated with chemical bonds, e.g. bond length, valence electron number, ionicity and *etc*. Strictly speaking, a pseudo-binary crystal represents a pair of neighboring atoms in the crystal, which is different from the traditional concept of a chemical bond, since complex bonding may form between atoms in a crystal. For example, both *σ* bond and *π* bond form between carbon atoms in graphite. Nevertheless, we will still call a couple of interacted neighboring atoms as a chemical bond for simplicity in the present work.

The hardness of a multi-component compound can be expressed as the geometrical average of hardness of all pseudo-binary crystals that comprise the compound^9^:


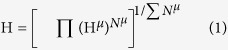


where H^*μ*^ and *N*^*μ*^ respectively represents the hardness and number of the *μ* type bond in the compound.

The hardness of *μ* type bond equals to the resistance of the bond per unit area to the indenter^10^:


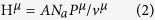


where *A* is a proportional coefficient, *N*_*a*_ is the covalent bond number per unit area, *P*^*μ*^ is the Mulliken overlap population^13^, and *v*^*μ*^ is the bond volume. In the equation, *P*^*μ*^/*v*^*μ*^ characterizes the strength of the bond, as suggested by Gao^10^. For a specific bond, the bond number per unit area *N*_*a*_ is evaluated from its electron number per cubic angstroms as (*N*_e_^*μ*^/2)^2/3^, where *N*_e_^*μ*^ equals to *n*^*μ*^/*v*^*μ*^ with *n*^*μ*^ being the number of electrons of the bond. Substitution *N*_*a*_ into equation (2), one obtains:


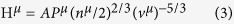


However, in the original work by Gao^10^, H^*μ*^ was expressed as:


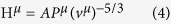


It reveals that equation (4) is over-simplified, since *n*^*μ*^ of a particular bond is not always 2. To make sure that the model is applicable to crystals comprised of complex bond types, further modifications are still necessary, which will respectively be taken on *n*^*μ*^, *v*^*μ*^ and the averaging process.

**Figure 1:**
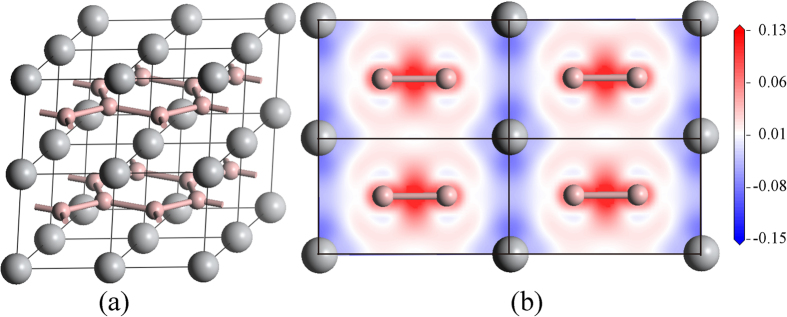
(**a**) Crystal structure of TiB_2_. (**b**) Electron density difference map, which represents charge redistribution due to formation of chemical bonds, on (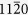) plane to illustrate the bonding nature of TiB_2_. The map displays the characteristics of B-B *σ* bond, B-B *π* bond and Ti-B ionic-covalent bond. For example, the strong B-B *σ* bond results in a substantial redistribution of electrons into the space between B-B, while the *π* bond is in accordance with the shoulder to shoulder type of electron redistribution.

Previously, the number of valence electrons *n*^*μ*^ of a bond follows^14^:


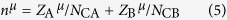


where *Z*_A_^*μ*^ and *N*_CA_ are the valence electron number and coordination number of the A atom constructing *μ* type bond, respectively. *Z*_B_^*μ*^ and *N*_CB_ are in analogous to *Z*_A_^*μ*^ and *N*_CA_. Equation (5) explicitly assumes that the valence electron of atom A is equally partitioned to bonds surrounding it. This assumption is only reasonable when bonds surrounding the atom are similar in nature. However, in a crystal comprised of complex bond types, the equal partition may result in an unrealistic *n*^*μ*^ that deviates significantly from the true valence electron number of the bond. Take TiB_2_ as an example. The electron redistribution map in Fig. 1b displays the characteristics of B-B *σ* bond, B-B *π* bond and Ti-B ionic-covalent bond^15^. In principle, each B-B *σ* bond contains 2 valence electrons and B atoms need extra electrons transferred from Ti to form *π* bond. The rest valence electrons of Ti form ionic-covalent bond between Ti and B. If we assume that valence electrons of Ti are equally partitioned to 3 B-B *π* bond and 12 Ti-B bond surrounding the Ti atom, then valence electron number for B-B and Ti-B bond is respectively 34/15 = 2 + 4/15 and 4/15. However, if we use equation (5), valence electron numbers for both bonds are 2/3 with *N*_B_ = 9 and *N*_Ti_ = 12. Obviously, the valence electron number derived from equation (5) is unrealistic, especially when bonds surrounding an atom are significantly different in nature, or electron transfer is involved during the formation of a crystal, or non-bonding electrons (lone pair electrons) exist.

The second modification is made on the definition of bond volume *v*^*μ*^. The original definition of bond volume is introduced by Levine^16^ in 1973, which assumes that the volume of a bond is proportional to (*d*^*μ*^)^3^. *d*^*μ*^ is the bond length. This definition has been broadly adopted in models associated with chemical bonds for over four decades. Here, we suggested that the bond volume (influence region of a bond) is not just correlated to its length, but also proportional to its valence electron number. Accordingly, the cell volume is partitioned to the component chemical bonds with *v*^*μ*^ being:


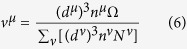


where Ω represents the cell volume of the crystal.

The third modification is conducted on the averaging process. In equation (1), the hardness is averaged over the number of bonds. Similar to the modification in bond volume, the valence electron number is also emphasized in the averaging process as follows:


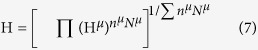


Both modification on bond volume and the average process emphasize the crucial role of valence electron populations, which means that both decomposing the crystal into pseudo-binary crystals and estimating properties of the crystal from properties of pseudo-binary crystals should be based on valence electron populations of different bonding states. Comparing to the purely geometrical considerations based on crystal structures, taking the population of valence electrons into consideration is more physical in nature, while it is well-known that properties of a crystal depend strongly on bonding states of valence electrons. For a deep understanding on the modifications, take *n*^*μ*^*N*^*μ*^ as a whole, which accounts for a group of valence electrons occupying the same electron bands. A set of specific electron bands of a crystal are comparable to molecular orbits of a specific chemical bond. In general, a well-defined chemical bond is occupied by 2 valence electrons. Then, we can define *M*^*μ*^ = *n*^*μ*^*N*^*μ*^/2 as the number of equivalent chemical bonds formed by these valence electrons. At this circumstance, the Mulliken overlap population of an equivalent bond is *Q*^*μ*^ = *N*^*μ*^*P*^*μ*^/*M*^*μ*^ = 2*P*^*μ*^/*n*^*μ*^. Substitution *Q*^*μ*^ and *M*^*μ*^ into equation (4) and the original definition of *v*^*μ*^ results in the hardness of the equivalent chemical bond H^*μ*^ being:


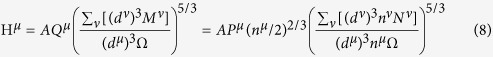


Equation (8) is exactly the same as equation (3) with the bond volume defined in equation (6). In addition, take equation (8) and *M*^*μ*^ into equation (1) results in equation (7). It indicates that the modifications are self-consistent and equivalent to decomposing the crystal into a set of well-defined chemical bonds. It is noteworthy that equation (8) reduces to equation (4) that was proposed in the original model^10^, when all the bonds in the crystal are well-defined chemical bond, i.e. 2 valence electrons per pseudo-binary crystal. According to equation (8), hard chemical bonds need high covalency of the bond (high 2*P*^*μ*^/*n*^*μ*^), short bond length and high valence electron density. These conditions are consistent with other microscopic models^9^,^10^,^11^,^12^, which can facilitate the quest for intrinsic super-hard materials.

## Evaluation of the Modified Model

Crystals from refs 7,9, 10, 11 are selected to check the availability of the modified model. These crystals are classified into three groups, which are corresponding to crystals with zinc blende or wurtzite structure, rock salt structure, and other complex structures. The results are listed in Tables 1, 2 and 3, respectively. The Mulliken overlap population *P*^*μ*^ of a bond was evaluated using first-principles calculations by CASTEP^17^. The Vanderbilt-type ultrasoft pseudopotential^18^ and exchange-correlation described by generalized gradient approximation^19^ were employed. The plane wave cutoff energy was set to be 500 eV. *k*-points mesh with a separation of 0.03 Å^−1^ according to Monkhorst-Pack method^20^ was adopted in the Brillouin zone. For each crystal, the structure was optimized and compared with experimental data to confirm the reliability of the calculation.

**Figure 2:**
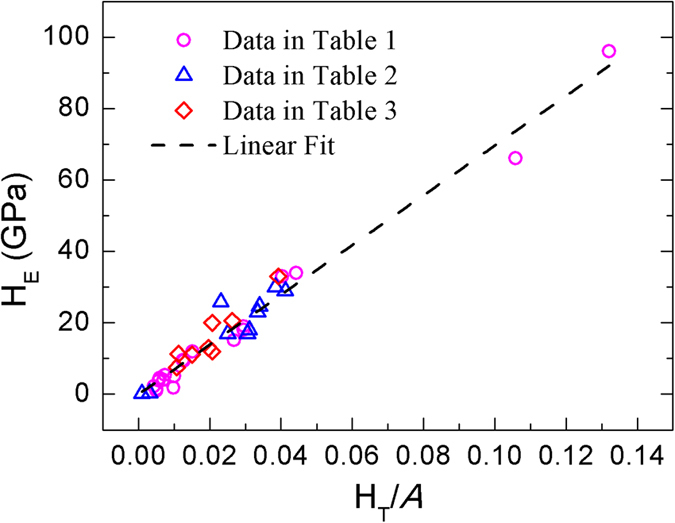
Linear fit of the modified model.

In order to determine the coefficient *A* in equation (4), theoretical values H_T_/*A* determined from crystal structures and bonding properties versus experimental hardness values H_E_ are plotted in Fig. 2. It is clear that all three sets of data locate at a straight line passing through the origin. By fitting, *A* is determined to be 693 with R^2^ = 0.984, which is close to the value of 740 suggested by Gao^10^ that was derived from the hardness value of diamond. The coincidence of *A* is not surprising, since the modified model reduces to the original one when *n*^*μ*^ of each bond equals to 2. For simplification, *A* is taken as 700 in the future. The theoretical hardness values derived from the modified model are also listed in Tables 1, 2 and 3 for comparison, where good agreement is obtained.

**Figure 3:**
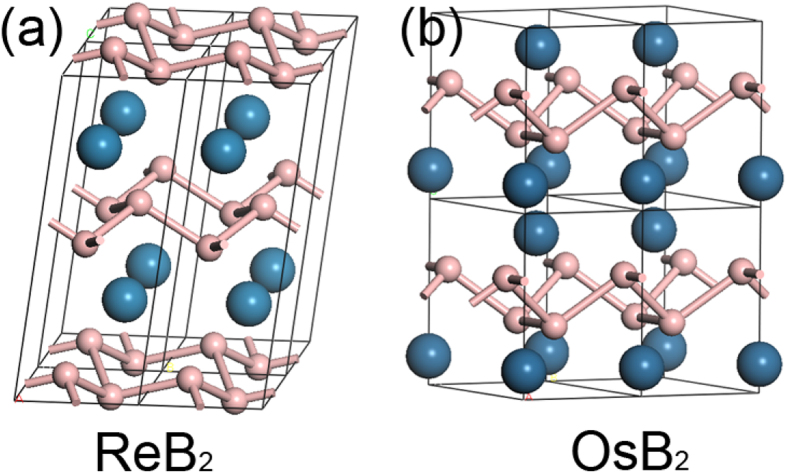
Crystal structure of ReB_2_ and OsB_2_.

To further verify the capability of the modified model, it is applied to investigate the hardness of TMB_2_s (TM = Ti, Zr, Hf, Re and Os). TiB_2_, ZrB_2_ and HfB_2_ have a simple hexagonal structure (space group P6/mmm), where TM and B atoms are respectively occupy 1a(0, 0, 0) and 2d(1/3, 2/3, 1/2) Wyckoff sites, as shown in Fig. 1a. ReB_2_ has a simple hexagonal structure (space group P6_3_/mmc), where Re and B atoms respectively occupy 2c(1/3, 2/3, 1/4) and 4f(1/3, 2/3, 0.548) Wyckoff sites, as shown in Fig. 3a. OsB_2_ has an orthorhombic structure (space group Pmmn), where Os and B atoms respectively occupy 2a(1/4, 1/4, 0.154) and 4f(0.058, 1/4, 0.632) Wyckoff sites, as shown in Fig. 3b. Different from crystals in Tables 1, 2 and 3, where chemical bonds are well-defined, decomposing these TMB_2_ into different kinds of pseudo-binary crystals is not intuitive. Analysis on the decomposition is guided by *P*^*μ*^. Any pair of atoms with positive *P*^*μ*^ is assumed to be an effective pseudo-binary crystal. Take ReB_2_ for example. According to *P*^*μ*^, there are four types of bonds in ReB_2_ (Table 4), B-B bonds, two types of Re-B bonds and Re-Re bonds. The B-B bonds are typical covalent *σ* bonds with 2 valence electrons per bond. To specify the electron number of other chemical bonds, it is assumed that valence electrons of Re is equally partitioned to all Re-B bonds and Re-Re bonds surrounding it. Therefore, each bond shares 1/2 electron from each Re atom, which means 1/2 electron per Re-B bond and 1 electron per Re-Re bond. In analogous, B-B bonds in OsB_2_ are assumed to be typical covalent *σ* bonds with 2 valence electrons, while Os-B bonds share valence electrons from Os resulting in 1 electron per bond. Decomposition of MB_2_ (M = Ti, Zr, Hf) has been introduced above during modification of *n*^*μ*^, which will not be repeated. With an appropriate allocation of valence electrons to the chemical bonds, the hardness can be predicted by the modified model. The predictions agree well with experimental measurements for these TMB_2_s, as shown in Table 4.

**Figure 4:**
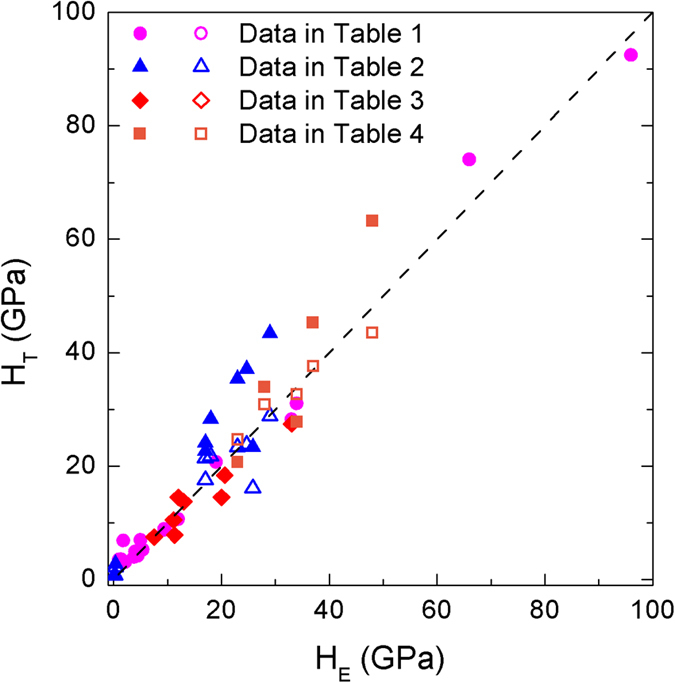
Calculated hardness values from models with and without modifications versus the experimental values. Solid points represent values calculated from the previous Gao’s model, while hollow points are calculated from the modified model. The dash line means H_T_ = H_E_.

To verify the improvement of the modifications, hardness values predicted by using the original Gao’s model and the modified model were compared with the experimental measurements, as shown in Fig. 4. In the calculation by using the original model, the proportional coefficient *A* is also adopted as 700 instead of 740. As stated above, when all the decomposed pseudo-binary crystals occupy 2 valence electrons, the modified model reduces to the original one. Therefore, results for crystals in Tables 1 and 3 obtained from both models are the same, as demonstrated in Fig. 4 that results predicted by different models overlap with each other. However, hardness values for crystals in Table 2 predicted by the modified model are lower than those from the original model. As shown in Fig. 4, without any modification, hardness values of transition metal carbides and nitrides predicted by the original model exhibit a systematic over-estimation. For a rock-salt structure crystal, its unit cell is decomposed into 6 equivalent pseudo-binary crystals with *n*^*μ*^ less than 2. Therefore, hardness values predicted by the modified model will be (*n*^*μ*^/2)^2/3^ times those predicted by the original model. After modification, the predicted values agree well with experiments. Figure 4 also reveals that the predicted hardness values for those TMB_2_s are also significantly improved after modification.

Before ending, some fundamental aspects on hardness are discussed. It should be noted that experimental measured hardness values usually exhibit significant divergence, since the measurements are very sensitive to many parameters, including loading and unloading speed, applied load, anisotropy of materials, defects in the sample, method of measurement, temperature, *etc*^4^. As a consequence, a great number of values on hardness are reported for each crystal, which makes selecting the reliable hardness value of a material a great challenge.

Though hardness tests are easy to conduct, interpretation on hardness values are complex. Usually, the experimental hardness value is found decreasing with increasing load, which is referred to as the size effect^21^. When the load reaches a certain level, the measured hardness value will not decrease anymore. This asymptotic value in the hardness-load curve is commonly recommended as the reliable hardness value of a hard and brittle material^22^. One question arising from the size effect is: will the hardness monotonously increases with the decrease of load and approaches infinite? Despite of the plateau at large loads in the hardness-load curve, another plateau was obtained at small loads during hardness measurements by Wang *et al.*^23^. As illustrated in their work, the asymptotic value associated with small loads is more or less a constant, while the asymptotic hardness value in accordance with large loads depends strongly on microstructures^23^. In addition, transition from the constant value to the trend of decreasing with increasing load was found coincident with the onset of cracking around the indentation^24^. It means that the constant hardness level obtained at small loads is probably the “intrinsic hardness” of a material, which is a measure of the resistance to plastic deformations without initiation of any micro-cracks. In contrast, the asymptotic hardness level at large loads is a complex composite of the resistance to plastic deformation and fracture with the microstructure saturated by micro-cracks. For simplification, the asymptotic hardness level at large loads is called “engineering hardness”.

While microscopic hardness models assume a perfect crystal, the predict hardness value should be close to the “intrinsic hardness” of a material, since only when the material is lightly deformed that the material can still be well characterized as a continuous crystal. Therefore, the predicted hardness values should be compared to hardness values measured at small loads instead of the asymptotic hardness level at large loads. There is no doubt that the “engineering hardness” of a material is a crucial property in its practical uses due to the severe service environment. Even though a material with high “intrinsic hardness” may display low “engineering hardness”, it is essential that a material with high “engineering hardness” should at least contain some components with high “intrinsic hardness”. Therefore, it is desirable to develop microscopic models to explore potential intrinsic hard materials.

## Conclusions

In the present work, three major modifications were introduced to the theoretical hardness model proposed by Gao^10^. After modification, the model predicts well the intrinsic harness values of many crystals, including those crystals composed of complex chemical bonds. The modifications are:

(1) The valence electron of a chemical bond should be specified based on its bonding nature instead of equally partitioning of valence electrons of an atom to its connecting bonds;

(2) The bond volume *v*^*μ*^ is not only proportional to the cubic power of bond length (*d*^*μ*^)^3^, but also proportional to its valence electron number *n*^*μ*^;

(3) Deriving the hardness of a crystal from the hardness values of chemical bonds should be averaged based on valence electron population.

All these modifications emphasize the crucial role of valence electron populations, which means that properties of a crystal depend strongly on bonding states of valence electrons. Both decomposing the crystal into pseudo-binary crystals and estimating properties of the crystal from properties of pseudo-binary crystals should be based on valence electron populations of different bonding states. The model becomes self-consistent by introducing these modifications, which is equivalent to decomposing a crystal to a set of well-defined chemical bonds with 2 valence electrons. The fundamental idea of these modifications may also be applicable to other models associated with chemical bonds, e.g. models to estimate thermal expansion^25^ or bulk modulus^26^,^27^. In general, derivations of these models usually start from simple crystals comprised of well-defined chemical bonds, such as crystals in Table 1. Exploring a self-consistent way to define equivalent chemical bonds may directly extend the models suitable for complex crystals.

## Additional Information

**How to cite this article**: Dai, F.-Z. and Zhou, Y. A Modified Theoretical Model of Intrinsic Hardness of Crystalline Solids. *Sci. Rep.* **6**, 33085; doi: 10.1038/srep33085 (2016).

## Figures and Tables

**Table 1:** Crystallographic features (including cell volume Ω, bond length *d*^*μ*^, bond number *N*^*μ*^, valence electron number per bond *n*^*μ*^ and bond volume *v*^*μ*^) and Mulliken bond overlap population *P*^*μ*^ of typical zinc blende and wurtzite structure crystals.

	Ω (Å^3^)	*d*^*μ*^ (Å)	*N*^*μ*^	*n*^*μ*^	*v*^*μ*^ (Å^3^)	*P*^*μ*^	H_T_/*A*	H_T_ (GPa)	H_E_ (GPa)
Dia	11.34	1.544	4	2	2.834	0.75	0.1321	92.5	96^a^
Si	40.77	2.366	4	2	10.193	0.73	0.0152	10.7	12^a^
*c*-BN	11.88	1.569	4	2	2.971	0.65	0.1058	74.1	66^a^
*β*-SiC	20.77	1.890	4	2	5.193	0.69	0.0443	31.0	34^a^
BP	23.11	1.958	4	2	5.778	0.75	0.0403	28.2	33^b^
AlP	41.62	2.382	4	2	10.405	0.63	0.0127	8.9	9.4^a^
GaP	41.60	2.382	4	2	10.399	0.62	0.0125	8.8	9.5^a^
InP	53.20	2.585	4	2	13.298	0.56	0.0075	5.3	5.4^a^
BAs	27.39	2.072	4	2	6.848	0.73	0.0296	20.7	19^b^
AlAs	46.99	2.480	4	2	11.747	0.61	0.0100	7.0	5^a^
InAs	59.28	2.680	4	2	14.819	0.51	0.0057	4.0	3.8^a^
AlSb	60.26	2.695	4	2	15.066	0.64	0.0070	4.9	4^a^
GaSb	59.62	2.685	4	2	14.906	0.54	0.0060	4.2	4.5^a^
InSb	72.86	2.871	4	2	18.216	0.55	0.0044	3.1	2.2^a^
ZnS	40.25	2.356	4	2	10.061	0.46	0.0098	6.9	1.8^a^
ZnSe	45.86	2.460	4	2	11.464	0.3	0.0051	3.6	1.4^a^
ZnTe	59.01	2.676	4	2	14.752	0.44	0.0050	3.5	1^a^
AlN	42.36	1.900	6	2	5.268	0.58	0.0295	20.7	18^a^
		1.912	2	2	5.375	0.26			
GaN	47.32	1.971	6	2	5.897	0.58	0.0268	18.8	15.1^a^
		1.979	2	2	5.968	0.37			

H_T_ and H_E_ are theoretical and experimental values of hardness, respectively.

^a^experimental data taken from ref. 7 and the references therein.

^b^experimental data taken from ref. 11 and the references therein.

**Table 2:** Crystallographic features (including cell volume Ω, bond length *d*^*μ*^, bond number *N*^*μ*^, valence electron number per bond *n*^*μ*^ and bond volume *v*^*μ*^) and Mulliken bond overlap population *P*^*μ*^ of typical rock salt structure crystals.

	Ω (Å^3^)	*d*^*μ*^ (Å)	*N*^*μ*^	*n*^*μ*^	*v*^*μ*^ (Å^3^)	*P*^*μ*^	H_T_/*A*	H_T_ (GPa)	H_E_ (GPa)
TiC	20.32	2.166	6	8/6	3.386	0.34	0.0340	23.8	24.7^a^
ZrC	26.04	2.352	6	8/6	4.340	0.35	0.0231	16.2	25.8^a^
VC	17.95	2.078	6	9/6	2.992	0.31	0.0412	28.8	29^a^
NbC	22.47	2.240	6	9/6	3.745	0.34	0.0311	21.8	18^a^
TiN	19.14	2.123	6	9/6	4.177	0.28	0.0334	23.4	23^a^
HfN	25.06	2.323	6	9/6	3.587	0.33	0.0251	17.6	17^a^
NbN	21.52	2.208	6	10/6	3.495	0.29	0.0306	21.4	17^a^
NaCl	46.04	2.845	6	8/6	11.509	0.12	0.0031	2.1	0.3^b^
KCl	63.43	3.165	6	8/6	15.859	0.07	0.0010	0.7	0.2^b^

H_T_ and H_E_ are theoretical and experimental values of hardness, respectively.

^a^experimental data taken from ref. 7 and the references therein.

^b^experimental data taken from ref. 11 and the references therein.

**Table 3:** Crystallographic features (including cell volume Ω, bond length *d*^*μ*^, bond number *N*^*μ*^, valence electron number per bond *n*^*μ*^ and bond volume *v*^*μ*^) and Mulliken bond overlap population *P*^*μ*^ of other complex crystals.

	Ω (Å^3^)	*d*^*μ*^ (Å)	*N*^*μ*^	*n*^*μ*^	*v*^*μ*^ (Å^3^)	*P*^*μ*^	H_T_/*A*	H_T_ (GPa)	H_E_ (GPa)
*α*-SiO_2_	121.63	1.614	6	2	10.115	0.53	0.0112	7.8	11^b^
		1.617	6	2	10.157	0.53			
SiO_2_ (Stishovite)	47.79	1.768	8	2	3.837	0.42	0.0392	27.4	33^a^
		1.833	4	2	4.273	0.34			
SnO_2_	78.45	2.116	8	2	6.525	0.36	0.0150	10.5	11.1^b^
		2.121	4	2	6.564	0.31			
TiO_2_	47.79	1.959	8	2	5.206	0.39	0.0207	14.5	12^b^
		2.003	4	2	5.571	0.25			
Al_2_O_3_	87.47	1.871	6	2	3.301	0.35	0.0207	14.5	20^a^
		1.993	12	2	3.988	0.26			
Y_2_O_3_	696.66	2.376	24	2	6.999	0.34	0.0107	7.5	7.5^a^
		2.393	24	2	7.152	0.32			
		2.404	24	2	7.252	0.30			
		2.445	24	2	7.624	0.22			
*m*-ZrO_2_	143.91	2.055	4	2	4.356	0.44	0.0196	13.7	13^a^
		2.081	4	2	4.522	0.34			
		2.163	4	2	5.080	0.30			
		2.166	4	2	5.104	0.28			
		2.173	4	2	5.152	0.24			
		2.271	8	2	5.882	0.26			
*c*-RuO_2_	115.44	1.993	24	2	4.810	0.36	0.0263	18.4	20^a^

H_T_ and H_E_ are theoretical and experimental values of hardness, respectively.

^a^experimental data taken from ref. 7 and the references therein.

^b^experimental data taken from ref. 10 and the references therein.

**Table 4:** Crystallographic features (including cell volume Ω, bond length *d*^*μ*^, bond number *N*^*μ*^, valence electron number per bond *n*^*μ*^ and bond volume *v*^*μ*^) and Mulliken bond overlap population *P*^*μ*^ of TMB_2_s.

	*a* (Å)	*b* (Å)	*c* (Å)	Ω (Å^3^)	*μ*	*d*^*μ*^ (Å)	*N*^*μ*^	*n*^*μ*^	*v*^*μ*^ (Å^3^)	*P*^*μ*^	H^*μ*^ (GPa)	H_T_ (GPa)	H_E_ (GPa)
TiB_2_	3.028	3.028	3.223	25.58	B-B	1.748	3	34/15	3.907	0.85	66.7	32.7	34^a^
					Ti-B	2.377	12	4/15	1.155	0.05	7.2		
ZrB_2_	3.167	3.167	3.540	30.75	B-B	1.828	3	34/15	4.518	0.85	52.4	24.7	23^b^
					Zr-B	2.545	12	4/15	1.433	0.05	5.0		
HfB_2_	3.163	3.163	3.516	30.47	B-B	1.826	3	34/15	4.497	0.88	54.6	30.9	28^b^
					Hf-B	2.535	12	4/15	1.415	0.09	9.2		
ReB_2_	2.911	2.911	7.482	54.90	B-B	1.827	6	2	2.143	0.70	137.6	43.5	48^c^
					Re-B	2.229	4	1/2	0.973	0.14	40.7		
					Re-B	2.260	12	1/2	1.014	0.31	84.2		
					Re-Re	2.911	6	1	4.330	0.06	2.3		
OsB_2_	4.688	2.882	4.083	55.17	B-B	1.825	2	2	2.639	0.69	95.9	37.6	37^d^
					B-B	1.898	4	2	2.967	0.56	64.0		
					Os-B	2.172	4	1	2.225	0.12	14.0		
					Os-B	2.203	8	1	2.320	0.28	30.4		
					Os-B	2.300	4	1	2.642	0.24	21.0		

H_T_ and H_E_ are theoretical and experimental values of hardness, respectively. H^*μ*^ represents the hardness of *μ* type bond.

^a^Ref. 28.

^b^Ref. 29.

^c^Ref. 30.

^d^Ref. 31.
